# The Relationship Between Trace Element Levels and Arginines in Individuals with Metabolic Syndrome: An Exploratory Cross-Sectional Analysis of Endocrine and Nitric Oxide Metabolism

**DOI:** 10.3390/jcm15218480

**Published:** 2026-10-30

**Authors:** Necip Nas, Muzaffer Aslan, Semih Saglik, Vugar Ali Turksoy

**Affiliations:** 1 Department of Internal Medicine, Siirt Training and Research Hospital, 56100 Siirt, Türkiye; 2 Department of Cardiology, Siirt Training and Research Hospital, 56100 Siirt, Türkiye; 3 Department of Radiology, Siirt Training and Research Hospital, 56100 Siirt, Türkiye; 4 Department of Public Health, Faculty of Medicine, Yozgat Bozok University, 66100 Yozgat, Türkiye

**Keywords:** metabolic syndrome, trace elements, arginine metabolism, endothelial dysfunction

## Abstract

**Background:** Metabolic syndrome (MetS) is characterized by a cluster of cardiometabolic abnormalities associated with endothelial dysfunction, oxidative stress, and chronic low-grade inflammation. Disturbances in the arginine–nitric oxide (NO) pathway and alterations in trace element homeostasis have independently been implicated in the pathogenesis of MetS. However, the associations between essential and potentially toxic trace elements and biomarkers of arginine metabolism remain poorly understood. This study aimed to investigate the associations between serum trace element concentrations and arginine metabolism biomarkers in individuals with metabolic syndrome. **Methods:** This cross-sectional study included 72 adults with MetS diagnosed according to International Diabetes Federation criteria. Serum concentrations of manganese (Mn), selenium (Se), copper (Cu), zinc (Zn), antimony (Sb), molybdenum (Mo), and thallium (Tl) were determined by inductively coupled plasma mass spectrometry (ICP-MS). Asymmetric dimethylarginine (ADMA), symmetric dimethylarginine (SDMA), N-monomethyl-L-arginine (L-NMMA), and citrulline were quantified in all samples by liquid chromatography-tandem mass spectrometry (LC-MS/MS), and all reported analyses are based on these LC-MS/MS values. The study was not powered for multivariable modeling, and the findings are therefore hypothesis-generating. **Results:** Citrulline correlated positively with Zn (ρ = 0.514, q < 0.001), Se (ρ = 0.428, q = 0.002), and Cu (ρ = 0.370, q = 0.008) and inversely with Mn (ρ = −0.505, q < 0.001). SDMA correlated inversely with Mn (ρ = −0.379, q = 0.007). The HC3-robust SDMA model was significant overall (R^2^ = 0.219, *p* = 0.005), whereas the adjusted Mn coefficient did not reach conventional statistical significance and its 95% CI included zero (B = −0.00475, 95% CI, −0.00955 to 0.00004; *p* = 0.052). An increase of one interquartile range in Mn corresponded to a predicted SDMA change of −0.0414 µmol/L (95% CI, −0.0832 to 0.0003). The citrulline model was significant overall (R^2^ = 0.271, *p* < 0.001). **Conclusions:** Zn, Se, Cu, and Mn were associated with citrulline and SDMA; however, the adjusted estimates were imprecise. These exploratory cross-sectional findings do not establish causality, specificity to MetS, or clinical benefit and require confirmation in controlled prospective studies.

## 1. Introduction

Metabolic syndrome (MetS) is the clustering of central obesity, insulin resistance, hypertension, dyslipidemia, and impaired glucose metabolism, and it now affects approximately one-quarter of the adult population worldwide [1,2,3]. Its clinical weight lies less in the individual components than in the accelerated progression they produce towards type 2 diabetes mellitus (T2DM), cardiovascular disease (CVD), chronic kidney disease, non-alcoholic fatty liver disease (NAFLD), and premature mortality [4,5]. The mechanisms that bind these components together remain incompletely defined, but endothelial dysfunction, oxidative stress, and chronic low-grade inflammation are consistently identified as the shared pathogenic axis [6,7,8]. Within that axis, reduced nitric oxide (NO) bioavailability is among the earliest and most consequential events. Nitric oxide is generated from L-arginine by endothelial nitric oxide synthase (eNOS) and governs vasodilation, platelet aggregation, leukocyte adhesion, smooth muscle proliferation, and insulin-mediated glucose uptake [9,10]; its loss therefore aggravates hypertension, insulin resistance, and atherogenesis simultaneously, and has been linked to adverse cardiometabolic outcomes in MetS [11,12].

Because NO itself is too short-lived for routine measurement, the activity of this pathway is assessed through circulating arginine derivatives. Asymmetric dimethylarginine (ADMA) and N-monomethyl-L-arginine (L-NMMA) competitively inhibit NOS and thereby reduce NO production [13,14]. Symmetric dimethylarginine (SDMA) does not inhibit the enzyme directly but restricts cellular arginine transport and is an established marker of cardiovascular and renal risk [15]. Elevated ADMA and SDMA concentrations have been reported in obesity, T2DM, hypertension, NAFLD, and MetS, indicating that disturbed arginine metabolism is a feature shared across cardiometabolic disorders [16,17,18,19]. Citrulline, released together with NO during the eNOS reaction, serves as an indirect index of pathway activity [20,21,22].

A second and largely parallel body of work has examined trace elements as determinants of metabolic health. Zinc (Zn), selenium (Se), copper (Cu), manganese (Mn), and molybdenum (Mo) act as cofactors in insulin signaling, antioxidant defense, mitochondrial function, and vascular regulation [23,24]. Zinc participates in insulin synthesis, storage, secretion, and receptor activation; selenium supports antioxidant selenoproteins such as glutathione peroxidases and thioredoxin reductases; and copper and manganese are required by the superoxide dismutases that protect endothelial cells from oxidative injury [25,26,27,28,29,30,31,32,33]. Altered concentrations of these elements have been described in obesity, insulin resistance, T2DM, and MetS [24,25,26,27,28,29,30,31,32,33,34]. In parallel, chronic exposure to potentially toxic elements such as antimony (Sb) and thallium (Tl) has been associated with oxidative stress, mitochondrial injury, endothelial dysfunction, and cardiovascular disease [35,36,37,38], so that essential and potentially toxic elements are best evaluated together rather than in isolation.

These two lines of evidence converge on the same mechanism. Reactive oxygen species lower NO bioavailability through eNOS uncoupling, accelerated NO degradation, and accumulation of methylated arginines, and a deficit of antioxidant trace elements would be expected to amplify each of these steps [29,30,31]. Indirect support comes from clinical populations characterized by chronic inflammation: methylated arginines are elevated in silicosis [39], trace element imbalance has been associated with the endothelial activation markers intercellular adhesion molecule-1 (ICAM-1) and vascular cell adhesion molecule-1 (VCAM-1) [40], and toxic metal burden has been related to oxidative stress and early biological markers of tissue injury [41,42].

What has not been established is whether the two sets of measurements track one another within the same individuals once MetS is present. Few studies have characterized essential and potentially toxic trace elements simultaneously with arginine-pathway biomarkers in this population, and the associations therefore remain insufficiently described. The present study was designed to address that specific question: in adults who already meet MetS criteria, are serum concentrations of Mn, Se, Cu, Zn, Sb, Mo, and Tl associated with concentrations of ADMA, SDMA, L-NMMA, and citrulline? We hypothesized that essential elements with antioxidant and insulin-related functions would be associated with biomarkers indicating preserved arginine–NO pathway activity. Because no comparison group without MetS was recruited and the sample size was fixed by consecutive recruitment, the study is framed throughout as an exploratory, hypothesis-generating analysis of within-cohort associations.

## 2. Materials and Methods

### 2.1. Study Design and Ethical Approval

This single-center, descriptive cross-sectional study examined the associations between serum trace element concentrations and biomarkers of arginine metabolism in adults with metabolic syndrome (MetS). No comparison group without MetS was recruited, so all analyses are within-cohort associations rather than case–control comparisons. The protocol was approved by the Siirt University Ethics Committee (Decision No: 2025/01/07/1; Date: 2 July 2025) and conducted in accordance with the Declaration of Helsinki. Written informed consent was obtained from every participant before enrollment.

### 2.2. Clinical and Anthropometric Assessment

Age, sex, smoking status, alcohol consumption, medication use, and medical history were recorded using a standardized questionnaire. Trained healthcare professionals measured body weight and height with participants in light clothing and without shoes, and body mass index (BMI) was calculated as weight divided by the square of height (kg/m^2^). Waist circumference was measured midway between the lowest rib and the iliac crest. Blood pressure was measured after at least 10 min of rest with a calibrated automated sphygmomanometer, and the mean of two consecutive readings was recorded.

### 2.3. Study Population

Adults aged 18–65 years attending the Internal Medicine and Endocrinology outpatient clinics between July 2025 and January 2026 were screened consecutively for eligibility. MetS was defined using the International Diabetes Federation (IDF) and harmonized criteria, requiring at least three of the following: increased waist circumference, elevated triglycerides, reduced high-density lipoprotein cholesterol, elevated blood pressure, and impaired fasting glucose or a previous diagnosis of type 2 diabetes mellitus. Exclusion criteria were acute or chronic infection, autoimmune or chronic inflammatory disease, active malignancy, pregnancy or lactation, chronic kidney disease with an estimated glomerular filtration rate below 60 mL/min/1.73 m^2^, chronic liver disease, surgery or trauma within the preceding three months, alcohol or substance use disorder, occupational heavy metal exposure, and current use of vitamin, mineral, amino acid, antioxidant, or nitric oxide-modulating supplements. Recruitment ran for the fixed study period rather than to a pre-specified target, and the final sample of 72 participants was therefore determined by the number of eligible consecutive outpatients rather than by an a priori power calculation.

### 2.4. Blood Sampling and Sample Preparation

Venous blood was drawn between 08:00 and 10:00 a.m. after an overnight fast of 8–12 h into trace-element-free vacuum tubes, centrifuged at 3500× *g* for 10 min at 4 °C, and the serum separated immediately and stored at −80 °C. Metal-free polypropylene consumables were used throughout sample handling, and samples were transported and processed under controlled cold-chain conditions. Serum aliquots were stored for a mean of 3–6 months before analysis and underwent no more than one freeze–thaw cycle.

### 2.5. Determination of Trace Element Levels

Serum manganese (Mn), selenium (Se), copper (Cu), zinc (Zn), antimony (Sb), molybdenum (Mo), and thallium (Tl) were determined by inductively coupled plasma mass spectrometry (iCAP Qc ICP-MS, Thermo Fisher Scientific, Waltham, MA, USA). One milliliter of serum was digested with 5 mL of ultrapure 65% HNO_3_ in a closed-vessel microwave digestion system at a maximum temperature of 180 °C for 20 min and diluted to a final volume of 10 mL with ultrapure deionized water. Quantification used multi-element calibration standards, with an 11-point calibration curve established for each analytical run. Quality assurance comprised reagent blanks, duplicate analyses, calibration verification standards, and a certified reference material (Seronorm™ Trace Elements Whole Blood L-2, Sero AS, Billingstad, Norway). All samples were analyzed in triplicate and mean concentrations were used in the statistical analyses.

### 2.6. Determination of Arginine Metabolism Biomarkers

Serum arginine, citrulline, ADMA, SDMA, and N-monomethyl-L-arginine (L-NMMA) were quantified by liquid chromatography-tandem mass spectrometry (LC-MS/MS) in all 72 participants; every descriptive, correlation, subgroup, and multivariable result reported here is based exclusively on these measurements, and no immunoassay-derived value entered any reported analysis. Analyses used a Shimadzu LC-20AD liquid chromatography system (Shimadzu Corporation, Tokyo, Japan) coupled to an Applied Biosystems MDS SCIEX API 3200 mass spectrometer (SCIEX, Framingham, MA, USA) in positive electrospray ionization mode, with a Phenomenex Luna C18 column (Phenomenex, Torrance, CA, USA) for chromatographic separation. For sample preparation, 100 µL of methanol containing d7-ADMA was added to 200 µL of serum; the mixture was centrifuged at 13,000 rpm for 10 min to precipitate proteins, and the supernatant was evaporated to dryness under nitrogen at 60 °C. The residue was dissolved in freshly prepared butanol containing 5% acetic chloride (*v*/*v*), incubated at 60 °C for 20 min, evaporated under nitrogen, and reconstituted in 100 µL of water–methanol (90:10, *v*/*v*) containing 0.1% formic acid; a 40 µL aliquot was injected. Multiple-reaction monitoring settings were optimized during preliminary continuous-infusion experiments. Intra- and interday coefficients of variation were 7.3% and 9.1%, respectively. All samples were run in duplicate, internal quality-control samples were included in every batch, and laboratory personnel were blinded to participant characteristics.

### 2.7. Biochemical Measurements

Fasting plasma glucose, glycated hemoglobin (HbA1c), total cholesterol, triglycerides, high-density lipoprotein cholesterol (HDL-C), low-density lipoprotein cholesterol (LDL-C), creatinine, urea, alanine aminotransferase (ALT), aspartate aminotransferase (AST), and high-sensitivity C-reactive protein (hs-CRP) were measured on an automated clinical chemistry analyzer in the central biochemistry laboratory. Estimated glomerular filtration rate (eGFR) was calculated using the CKD-EPI equation.

### 2.8. Statistical Analysis

Analyses were performed in IBM SPSS Statistics version 26.0 (IBM Corp., Armonk, NY, USA) and R version 4.4.0 (R Foundation for Statistical Computing, Vienna, Austria). Normality was assessed with the Shapiro–Wilk test and visual inspection of histograms and Q–Q plots. Continuous variables are reported as mean ± standard deviation (SD) and as median [interquartile range, IQR]; categorical variables as frequencies and percentages. Because several variables departed from normality, associations between trace elements and arginine-metabolism biomarkers were quantified with Spearman’s rank correlation coefficient, and the resulting 28 *p*-values were adjusted with the Benjamini–Hochberg false discovery rate (FDR) procedure, with q < 0.05 considered significant. Two secondary descriptive analyses were pre-specified: biomarker distributions below versus at or above each element’s sample median were compared with the Mann–Whitney U test under the same FDR correction, and distributions across Mn quartiles were compared with the Kruskal–Wallis test, with focused Q1-versus-Q4 contrasts reported without multiplicity adjustment. Adjusted associations were examined with multivariable linear regression for ADMA, SDMA, L-NMMA, and citrulline; each model included age, BMI, and all seven trace elements simultaneously. Unstandardized (B) and standardized (β) coefficients, 95% confidence intervals, and *p*-values were obtained from HC3 heteroscedasticity-robust covariance estimates, and model fit was summarized by R^2^, adjusted R^2^, and the HC3 joint F test. Established MetS components (waist circumference, blood pressure, fasting glucose, and lipid parameters) were not entered as covariates because they defined cohort eligibility and were expected to be collinear with one another and with the trace element predictors. Variance inflation factors (VIFs) were calculated to assess collinearity; no stepwise or penalized variable-selection procedure was applied. A two-sided post hoc sensitivity analysis was performed in G*Power 3.1 (Heinrich-Heine-Universität Düsseldorf, Düsseldorf, Germany; α = 0.05, power = 80% [1 − β = 0.80], effect size = 0.3348, critical t = 1.99). All tests were two-tailed and *p* < 0.05 was considered statistically significant.

## 3. Results

Seventy-two adults with metabolic syndrome were analyzed. Mean age was 41.57 ± 8.90 years and mean BMI was 26.32 ± 7.14 kg/m^2^. Mean serum trace element concentrations were 7.79 ± 4.35 µg/L for Mn, 53.46 ± 10.00 µg/L for Se, 687.39 ± 149.96 µg/L for Cu, 853.22 ± 347.04 µg/L for Zn, 1.61 ± 0.33 µg/L for Sb, 0.70 ± 0.21 µg/L for Mo, and 2.23 ± 0.33 µg/L for Tl. Mean arginine-pathway biomarker concentrations were 0.89 ± 0.24 µmol/L for ADMA, 0.37 ± 0.04 µmol/L for SDMA, 0.03 ± 0.02 µmol/L for L-NMMA, and 22.11 ± 11.16 µmol/L for citrulline. Medians, interquartile ranges, and observed ranges for all variables are given in Table 1.

Set against the external comparison values listed in Table 1, mean selenium (53.46 µg/L) and copper (687.39 µg/L) concentrations fell below the corresponding ranges (>70 µg/L and 850–1900 µg/L), mean thallium (2.23 µg/L) exceeded the upper value (0–2 µg/L), and mean manganese, zinc, antimony, and molybdenum concentrations lay within their respective intervals. These comparison values derive from occupational and toxicological guidance documents rather than from nutritional reference intervals for the general Turkish population and are reported here for descriptive orientation only.

The Shapiro–Wilk test showed no significant departure from normality for Se, Tl, or ADMA (*p* > 0.05), whereas Mn, Cu, Zn, Sb, Mo, SDMA, L-NMMA, and citrulline were non-normally distributed (*p* < 0.05); Spearman correlations were therefore used. Five of the 28 correlations remained significant after FDR correction (Table 2; Figure 1). Citrulline was positively associated with Zn (ρ = 0.514, q < 0.001), Se (ρ = 0.428, q = 0.002), and Cu (ρ = 0.370, q = 0.008), and inversely associated with Mn (ρ = −0.505, q < 0.001). SDMA was inversely associated with Mn (ρ = −0.379, q = 0.007). Scatterplots of these five associations are shown in Figure 2. No ADMA or L-NMMA association survived correction, and the nominal Zn–SDMA association (*p* = 0.026) did not survive FDR control (q = 0.104).

The secondary median-split analyses reproduced the same pattern (Supplementary Table S2). Participants with Mn at or above the sample median (9.12 µg/L) had lower SDMA (q = 0.050) and lower citrulline (q = 0.001) than those below it, and participants with Se or Zn at or above their medians had higher citrulline (q = 0.009 and q = 0.007, respectively). The Cu–citrulline contrast was nominally significant (*p* = 0.010) but narrowly missed FDR significance (q = 0.053). Across Mn quartiles, SDMA differed overall (Kruskal–Wallis H = 10.28, *p* = 0.016), declining from a median of 0.390 µmol/L in Q1 to 0.355 µmol/L in Q4, and citrulline also differed (H = 19.47, *p* < 0.001), from 26.378 µmol/L in Q1 to 13.915 µmol/L in Q4 (Supplementary Table S1). The corresponding unadjusted Q1-versus-Q4 comparisons were *p* = 0.003 for SDMA and *p* < 0.001 for citrulline.

In the models adjusted simultaneously for age, BMI, and all seven trace elements, the HC3 joint tests were significant for SDMA (R^2^ = 0.219, *p* = 0.005) and citrulline (R^2^ = 0.271, *p* < 0.001) but not for ADMA (R^2^ = 0.168, *p* = 0.174) or L-NMMA (R^2^ = 0.187, *p* = 0.266) (Table 3). In the SDMA model the manganese coefficient was B = −0.00475 µmol/L per µg/L (95% CI, −0.00955 to 0.00004; standardized β = −0.587; *p* = 0.052), so the confidence interval included zero and manganese was not an independently significant predictor at the conventional threshold; an increase in manganese equal to the sample IQR (8.72 µg/L) corresponded to a predicted SDMA change of −0.0414 µmol/L (95% CI, −0.0832 to 0.0003). In the citrulline model no individual predictor reached *p* < 0.05 despite the significant joint test. Variance inflation factors ranged from 1.04 to 4.30, the highest being those of Mn (4.30) and Zn (3.98), indicating moderate but not severe shared variance. Complete coefficients are provided in Supplementary Table S3.

## 4. Discussion

The present study investigated the associations between serum trace element concentrations and biomarkers of arginine metabolism in individuals with metabolic syndrome (MetS), focusing on potential links between micronutrient homeostasis and the nitric oxide (NO) pathway. The principal findings were that citrulline concentrations were positively associated with zinc, selenium, and copper levels, whereas manganese exhibited significant inverse associations with both SDMA and citrulline. The overall HC3-robust SDMA model was significant, but the adjusted manganese coefficient narrowly missed the conventional significance threshold (*p* = 0.052). To our knowledge, this is among the first studies to simultaneously evaluate essential and potentially toxic trace elements together with ADMA, SDMA, L-NMMA, and citrulline in individuals with metabolic syndrome. Taken together, these findings indicate associations between trace element status and the arginine–NO pathway but do not establish an effect on endothelial dysfunction in MetS. Metabolic syndrome is characterized by a complex interaction of insulin resistance, central obesity, oxidative stress, chronic low-grade inflammation, and endothelial dysfunction, all of which contribute to increased cardiovascular morbidity and mortality [6,7,8]. Endothelial dysfunction is increasingly recognized as a central pathophysiological mechanism linking obesity, hypertension, dyslipidemia, and impaired glucose metabolism [6,11]. Nitric oxide represents one of the most important mediators of endothelial homeostasis, regulating vascular tone, platelet aggregation, leukocyte adhesion, smooth muscle proliferation, and insulin-mediated glucose uptake [9,10,11,12]. Therefore, disturbances in the arginine–NO pathway have attracted considerable attention as potential contributors to the development and progression of cardiometabolic disorders [13,14,15,16,17,18,19,20]. The descriptive analysis identified differences relative to selected external comparison ranges. Mean selenium and copper concentrations were below commonly accepted reference ranges, whereas thallium concentrations exceeded reference values [43,44,45]. These observations are consistent with previous studies reporting altered trace element homeostasis in obesity, insulin resistance, metabolic syndrome, and cardiovascular disease [23,24,28,29,30,31]. Lower selenium status may impair glutathione peroxidase activity, thereby increasing oxidative stress and reducing protection against reactive oxygen species [28,29,30]. Similarly, lower copper status may compromise Cu/Zn-superoxide dismutase activity and contribute to endothelial injury through impaired antioxidant defense [31]. Such disturbances may play an important role in the progression of endothelial dysfunction and cardiometabolic complications in MetS.

Although ADMA is widely regarded as one of the most important endogenous inhibitors of nitric oxide synthase, no statistically significant associations were observed between ADMA concentrations and any of the measured trace elements after correction for multiple testing. This finding may initially appear unexpected, given the established role of ADMA in endothelial dysfunction and cardiovascular disease [13,14,15,16,17,18,19]. However, ADMA metabolism is predominantly regulated by dimethylarginine dimethylaminohydrolase (DDAH), an enzyme highly sensitive to oxidative stress and inflammatory signaling [13,14]. Consequently, ADMA concentrations may be influenced more strongly by systemic inflammatory burden and metabolic disturbances than by variations in trace element status alone. It should be emphasized that neither inflammatory cytokines nor direct oxidative stress markers were measured in the present study; therefore, this explanation is offered as a plausible, hypothesis-generating interpretation rather than a data-supported mechanism and should be tested directly in future studies that incorporate inflammatory and oxidative stress biomarkers. Another possible explanation is that all participants already had metabolic syndrome, resulting in a relatively homogeneous degree of endothelial dysfunction and thereby limiting variability in ADMA concentrations. Interestingly, this observation differs from findings obtained in our previous study involving patients with silicosis, where significantly elevated ADMA, SDMA, and L-NMMA concentrations were observed compared with controls [39]. The discrepancy between the two studies may reflect disease-specific mechanisms influencing nitric oxide metabolism. While silicosis is characterized by severe pulmonary inflammation and fibrosis, metabolic syndrome represents a more complex systemic metabolic disorder. These differences suggest that chronic inflammatory diseases may affect methylated arginine metabolism through distinct biological pathways. Among all arginine metabolites evaluated, citrulline demonstrated the strongest associations with trace element concentrations. Citrulline was positively correlated with zinc, selenium, and copper, while exhibiting a strong inverse correlation with manganese. Because citrulline is generated during nitric oxide synthesis from L-arginine, it is widely considered an indirect marker of NO pathway activity [20,21]. Therefore, the observed relationships suggest that trace element homeostasis may influence endothelial nitric oxide production and vascular function.

An additional observation emerged from the descriptive comparison of measured concentrations with external reference values. According to the ACGIH-based reference values and previously published population data, mean selenium (53.46 µg/L) and copper (687.39 µg/L) concentrations in our cohort were below recommended physiological ranges, whereas mean thallium concentrations (2.23 µg/L) exceeded the suggested upper reference level [43,44,45]. In contrast, average manganese, zinc, antimony, and molybdenum concentrations generally remained within their respective reference intervals. Within this cohort, the descriptive comparisons indicated lower mean selenium and copper concentrations and a higher mean thallium concentration relative to the selected external values. The lower mean selenium concentration is particularly noteworthy because selenium is a critical component of antioxidant selenoproteins, including glutathione peroxidases and thioredoxin reductases [28,29,30]. Reduced selenium availability may impair antioxidant defenses, promote oxidative stress, and accelerate nitric oxide degradation. Therefore, the lower mean selenium concentration observed in our cohort may contribute to the chronic inflammatory and oxidative environment characteristic of metabolic syndrome. Similarly, the mean copper concentration was below the lower external reference value. Although copper excess has traditionally received greater attention in cardiovascular research, inadequate copper availability may impair Cu/Zn-superoxide dismutase activity and compromise endothelial antioxidant capacity [31]. The positive association between copper and citrulline observed in our study further supports a possible link between copper status and the regulation of nitric oxide metabolism. Interestingly, despite zinc concentrations being largely within the reference range, zinc demonstrated the strongest positive correlation with citrulline. This finding suggests that even within physiologically acceptable ranges, variations in zinc status may influence nitric oxide pathway activity and endothelial function. Thus, biological effects of zinc may not be restricted to overt deficiency states but may also operate across the normal physiological spectrum. Another notable finding was a mean thallium concentration above the selected external value. Thallium is a highly toxic environmental metal associated with mitochondrial dysfunction, oxidative stress, and cardiovascular toxicity [35,36,37,38,44]. Although no significant association was observed between thallium and arginine metabolites, the mean concentration above the selected external value warrants assessment in future exposure studies. The absence of significant correlations suggests that thallium may exert its adverse cardiometabolic effects through mechanisms other than direct modulation of the arginine–nitric oxide pathway. Alternatively, the relatively narrow range of thallium concentrations may have limited the ability to detect such associations. Collectively, these descriptive comparisons suggest that trace element status warrants further investigation in MetS. Because the study lacked a metabolically healthy comparison group, the observed concentrations cannot establish MetS-specific deficiencies, excess exposure, or clinical implications. Furthermore, the external values against which these concentrations were compared are drawn from occupational and toxicological guidelines rather than from nutritional reference intervals established for the general Turkish population; the apparently low mean selenium and copper and the elevated mean thallium concentrations should therefore be regarded as descriptive signals requiring confirmation against population-based nutritional reference data rather than as evidence of nutritional deficiency or toxic exposure.

The strongest positive association was observed between zinc and citrulline concentrations (ρ = 0.514, q < 0.001). Zinc is an essential trace element involved in insulin synthesis, secretion, receptor activation, antioxidant defense, immune regulation, and cellular signaling [25,26,27]. Experimental and clinical studies have demonstrated that zinc deficiency promotes oxidative stress, inflammatory activation, endothelial dysfunction, and impaired glucose metabolism [25,26,27]. Zinc has also been shown to stabilize endothelial nitric oxide synthase and preserve endothelial integrity. Therefore, the positive association between zinc and citrulline observed in our study is consistent with the hypothesis that adequate zinc status contributes to the maintenance of nitric oxide bioavailability and vascular homeostasis in individuals with metabolic syndrome. Similarly, selenium exhibited a significant positive association with citrulline concentrations (ρ = 0.428, q = 0.002). Selenium-containing enzymes, particularly glutathione peroxidases and thioredoxin reductases, constitute major components of the cellular antioxidant defense system [28,29,30]. Oxidative stress promotes nitric oxide degradation, endothelial dysfunction, and eNOS uncoupling, ultimately reducing NO bioavailability [10,12]. Several epidemiological studies have reported lower selenium concentrations in individuals with obesity, metabolic syndrome, and cardiovascular disease [28,29,30]. Consequently, the positive association between selenium and citrulline may indicate that selenium-mediated antioxidant protection contributes to the preservation of nitric oxide metabolism and endothelial function.

Copper also demonstrated a significant positive association with citrulline levels (ρ = 0.370, q = 0.008). Although copper excess may exert pro-oxidant effects, physiological copper concentrations are essential for mitochondrial respiration, cellular energy production, and antioxidant defense mechanisms [31]. Copper-dependent Cu/Zn-superoxide dismutase is one of the most important antioxidant enzymes protecting endothelial cells against oxidative injury. Therefore, the positive association between copper and citrulline observed in our cohort may reflect improved redox balance and preservation of nitric oxide signaling in individuals with adequate copper status. Interestingly, our previous study evaluating selenium, zinc, and copper status in euthyroid nodular goiter also emphasized the biological importance of these trace elements in endocrine and metabolic regulation [45].

The magnitude of this association (ρ = 0.370) is best interpreted in terms of copper-dependent redox stability rather than as a direct stoichiometric link between the two analytes. Because the conversion of citrulline to arginine and subsequently to nitric oxide requires a stable redox environment, the functional yield of circulating citrulline may be diminished even when its serum concentration remains within the usual range. Copper is required by Cu/Zn-superoxide dismutase and by cytochrome-c oxidase, and the activity of these copper-dependent systems is expected to decline before citrulline itself falls; a moderate positive correlation is therefore the pattern that would be anticipated if copper status governed the efficiency with which the arginine–nitric oxide pathway operates rather than the absolute concentration of its intermediates. In metabolic syndrome and non-alcoholic fatty liver disease, reduced serum copper has been attributed to impaired ceruloplasmin synthesis and hepatic copper insufficiency, which increase oxidative stress and accelerate nitric oxide quenching [46]. Within this framework, the combination of a normal citrulline concentration and a low copper concentration may itself indicate endothelial vulnerability: serum copper bioavailability has been reported to predict early vascular remodeling, including carotid intima–media thickening, in obese patients with hepatic steatosis and a low prevalence of comorbidities [46]. The copper–citrulline association observed in the present cohort is consistent with such a shared metabolic–vascular axis, in which copper deficiency weakens nitric oxide stability and citrulline tracks that decline, potentially marking the transition from metabolic syndrome to non-alcoholic fatty liver disease-associated endothelial dysfunction. Because ceruloplasmin, hepatic steatosis, and carotid intima–media thickness were not assessed here, this mechanistic account is offered as an interpretive framework consistent with the observed correlation rather than as a relationship demonstrated by the present data.

A further notable finding was the inverse association between manganese and SDMA concentrations. SDMA has emerged as an important biomarker of cardiovascular and renal risk and indirectly impairs nitric oxide production by reducing cellular arginine transport [15,19]. Elevated SDMA concentrations have been associated with endothelial dysfunction, inflammation, atherosclerosis, and adverse cardiovascular outcomes [15,19]. In the present study, manganese showed an inverse bivariate correlation with SDMA (ρ = −0.379, q = 0.007). In the multivariable model, however, the adjusted coefficient did not reach conventional statistical significance (B = −0.00475 µmol/L per µg/L; 95% CI, −0.00955 to 0.00004; standardized β = −0.587; *p* = 0.052). This pattern warrants further investigation into the potential role of manganese in the regulation of arginine metabolism and endothelial function. An increase of one interquartile range in manganese corresponded to a predicted SDMA change of −0.0414 µmol/L (95% CI, −0.0832 to 0.0003). Because the confidence interval included zero, the adjusted estimate remains imprecise and should not be interpreted as clinically meaningful without confirmation in larger studies. A potential biological explanation for this pattern relates to the role of manganese as an essential cofactor for mitochondrial manganese superoxide dismutase (MnSOD), a major antioxidant enzyme within mitochondria [23,24]. MnSOD protects cells against oxidative damage by detoxifying superoxide radicals and preserving mitochondrial integrity. Experimental evidence suggests that oxidative stress may increase accumulation of methylated arginines and promote endothelial dysfunction [18,19]. Therefore, lower manganese availability may facilitate oxidative stress-mediated increases in SDMA concentrations, whereas adequate manganese status may help preserve nitric oxide signaling. The direction of the adjusted estimate, although not statistically significant, is compatible with the possibility that manganese participates directly or indirectly in the regulation of endothelial homeostasis. An additional noteworthy observation was the strong inverse correlation between manganese and citrulline concentrations (ρ = −0.505, q < 0.001). Although the exact mechanism remains uncertain, this finding may reflect complex interactions between mitochondrial metabolism, oxidative stress, and nitric oxide synthesis. Because citrulline is generated during NO production, alterations in manganese status may influence the balance between oxidative and nitrosative pathways. Further mechanistic studies are required to determine whether manganese directly regulates citrulline metabolism or whether this association reflects broader disturbances in metabolic homeostasis.

In contrast to zinc, selenium, copper, and manganese, no correlations between antimony, molybdenum, or thallium and the arginine metabolites remained significant after correction for multiple testing. Molybdenum functions primarily as a cofactor for oxidase enzymes and is involved in sulfur amino acid metabolism [32,33]. However, evidence linking molybdenum directly to nitric oxide metabolism remains limited. Similarly, despite a higher mean thallium concentration relative to the selected external value, no FDR-significant correlations with ADMA, SDMA, L-NMMA, or citrulline were observed. Thallium is a well-recognized environmental toxicant associated with oxidative stress, mitochondrial dysfunction, and cardiovascular toxicity [35,36,37,38,44]. Nevertheless, the variability of thallium exposure within our cohort may not have been sufficient to influence arginine metabolism, or alternative biological pathways may mediate its adverse effects. Growing evidence suggests that environmental metals contribute to cardiometabolic disease through mechanisms involving oxidative stress, endothelial dysfunction, inflammation, and mitochondrial injury [35,36,37,38]. The American Heart Association recently identified contaminant metals as emerging cardiovascular risk factors [37]. Although the potentially toxic elements were not significantly associated with arginine metabolites in our study, the observed associations between essential trace elements and nitric oxide pathway biomarkers support the broader concept that micronutrient homeostasis and environmental exposures jointly influence vascular health. Our findings are also supported by previous investigations conducted by our research group. In patients with silicosis, we demonstrated significantly altered concentrations of ADMA, SDMA, and L-NMMA, supporting the role of nitric oxide pathway dysregulation in chronic inflammatory disorders [39]. Furthermore, in hemodialysis patients, trace element imbalances were associated with endothelial activation markers including ICAM-1 and VCAM-1 [40]. Taken together, these observations suggest that oxidative stress, endothelial dysfunction, trace element imbalance, and altered arginine metabolism represent interconnected biological pathways across a spectrum of chronic diseases. Because endothelial dysfunction precedes overt cardiovascular disease and may be reversible during the early stages of MetS [6,11,12], the observed biomarker associations may help inform future mechanistic studies. However, the present cross-sectional, single-cohort design does not support clinical risk prediction or targeted nutritional interventions. Prospective studies are needed to determine whether combined assessment of trace element status and arginine metabolites adds clinical value.

Several limitations should be acknowledged. First, the cross-sectional design precludes causal inference and prevents determination of temporal relationships. Second, the relatively modest sample size may limit the ability to detect weaker associations. The sample size reflects the number of eligible participants recruited consecutively during the study period rather than an a priori power calculation. Although exploratory median-split and quartile analyses were included, the modest sample size limited their precision, and focused post hoc contrasts were not adjusted for multiplicity. Third, dietary intake, supplement use, environmental exposures, and medication history were not comprehensively assessed and may have influenced trace element concentrations. Fourth, direct measurements of endothelial function, oxidative stress biomarkers, and inflammatory cytokines were not available. Because ADMA metabolism is particularly sensitive to inflammatory and oxidative stress pathways, the absence of these markers limits our ability to explain why ADMA showed no significant association with trace elements after FDR correction, and the explanation offered above should be regarded as hypothesis-generating rather than confirmed by the present data. Finally, because all participants had metabolic syndrome, comparisons with healthy control subjects could not be performed. Consequently, the present findings describe within-cohort associations among individuals with metabolic syndrome and should not be interpreted as case–control comparisons or as evidence of a causal or bidirectional relationship between trace elements and arginine metabolism; larger studies incorporating a metabolically healthy comparison group are needed to determine whether the observed alterations are specific to metabolic syndrome. In addition, the multivariable regression models included age, BMI, and trace elements as covariates but did not incorporate other metabolic syndrome components (waist circumference, blood pressure, glucose, and lipid parameters), because these variables define metabolic syndrome status itself and were considered likely to introduce circularity and collinearity; variance inflation factors were calculated, but penalized and stepwise variable-selection procedures were not performed. Moreover, the study was not powered for multivariable modeling, so the adjusted coefficients should be interpreted as hypothesis-generating rather than as precise effect estimates, and the external comparison values presented in Table 1 derive from occupational and toxicological guidelines rather than from population-based nutritional reference intervals for Turkish adults. Despite these limitations, the study has several strengths. To our knowledge, this is among the first investigations to simultaneously evaluate serum concentrations of Mn, Se, Cu, Zn, Sb, Mo, and Tl together with ADMA, SDMA, L-NMMA, and citrulline in individuals with metabolic syndrome. The combined assessment of micronutrient status and arginine metabolism provides exploratory evidence of associations between trace element status and biomarkers related to nitric oxide metabolism. In addition, ceruloplasmin, hepatic steatosis status, and carotid intima–media thickness were not measured, so the copper-related mechanistic interpretation outlined above could not be tested directly within this cohort and requires confirmation in studies that combine trace element, hepatic, and vascular phenotyping.

## 5. Conclusions

The objective of this study was to determine whether serum concentrations of essential and potentially toxic trace elements are associated with biomarkers of arginine metabolism in adults with metabolic syndrome. Within this cohort of 72 participants, the answer was affirmative for four of the seven elements and for two of the four biomarkers. Citrulline was positively associated with zinc, selenium, and copper and inversely associated with manganese, and SDMA was inversely associated with manganese; the same ordering was reproduced in the median-split and manganese-quartile analyses. No association involving ADMA, L-NMMA, antimony, molybdenum, or thallium survived correction for multiple testing.

After simultaneous adjustment for age, BMI, and all measured trace elements, the joint models for SDMA and citrulline remained significant, but no individual element emerged as an independent predictor: the adjusted manganese–SDMA coefficient approached the conventional threshold and its confidence interval included zero. The data therefore support an association between essential trace element status and the arginine–nitric oxide pathway in metabolic syndrome, while showing that they are not sufficient to isolate the contribution of any single element. Read within the scope of a cross-sectional, single-cohort design, these results accomplish what an exploratory analysis is intended to accomplish: they reduce a broad field of candidate relationships to a small and well-defined set worth testing directly. They identify manganese, zinc, selenium, and copper, and the citrulline and SDMA readouts in particular, as the most informative targets for larger controlled prospective studies incorporating direct measures of endothelial function, oxidative stress, and inflammatory status.

## Figures and Tables

**Figure 1:**
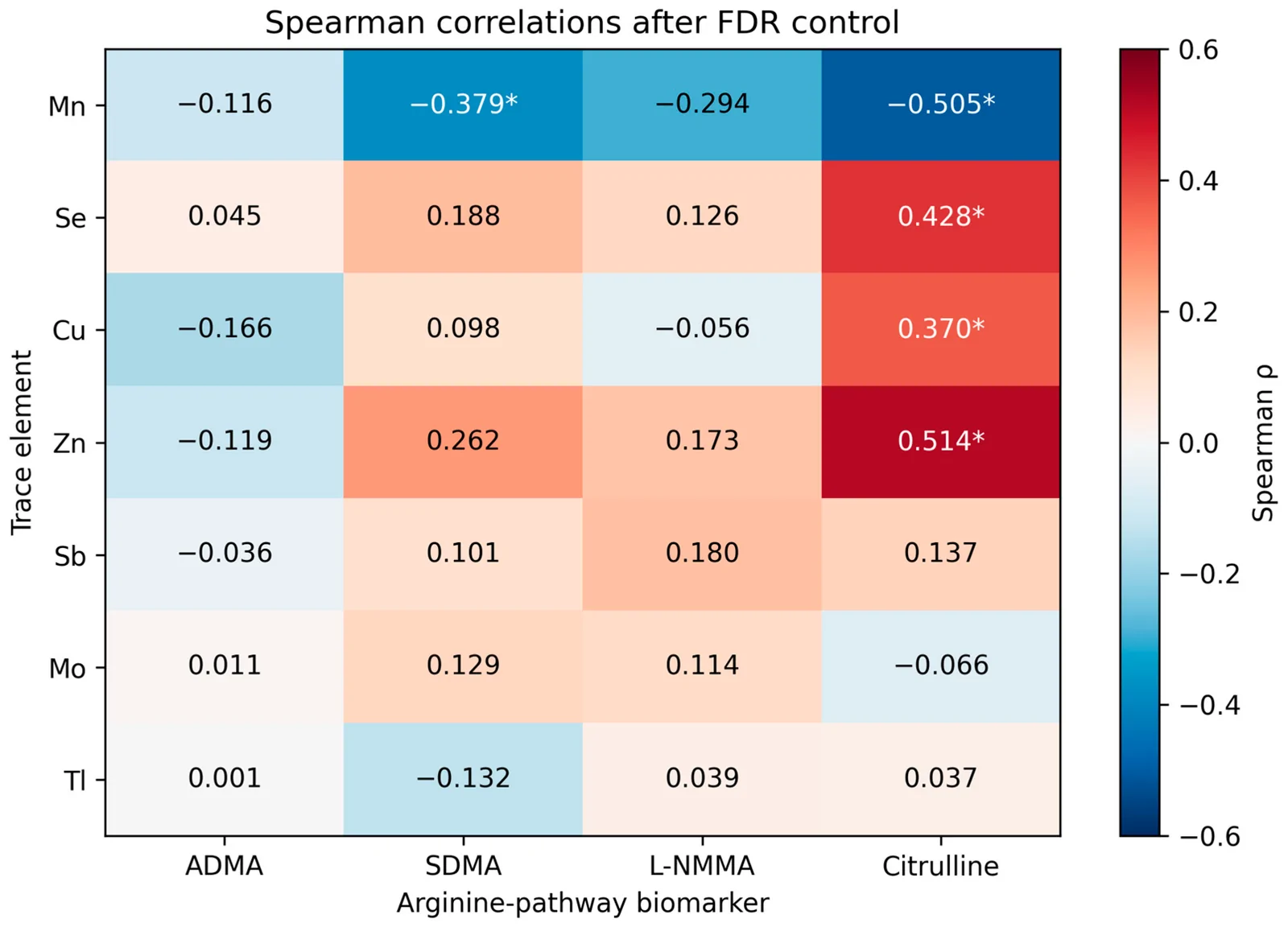
Correlation heatmap. Values are Spearman ρ; asterisks denote q < 0.05 after Benjamini–Hochberg correction.

**Figure 2:**
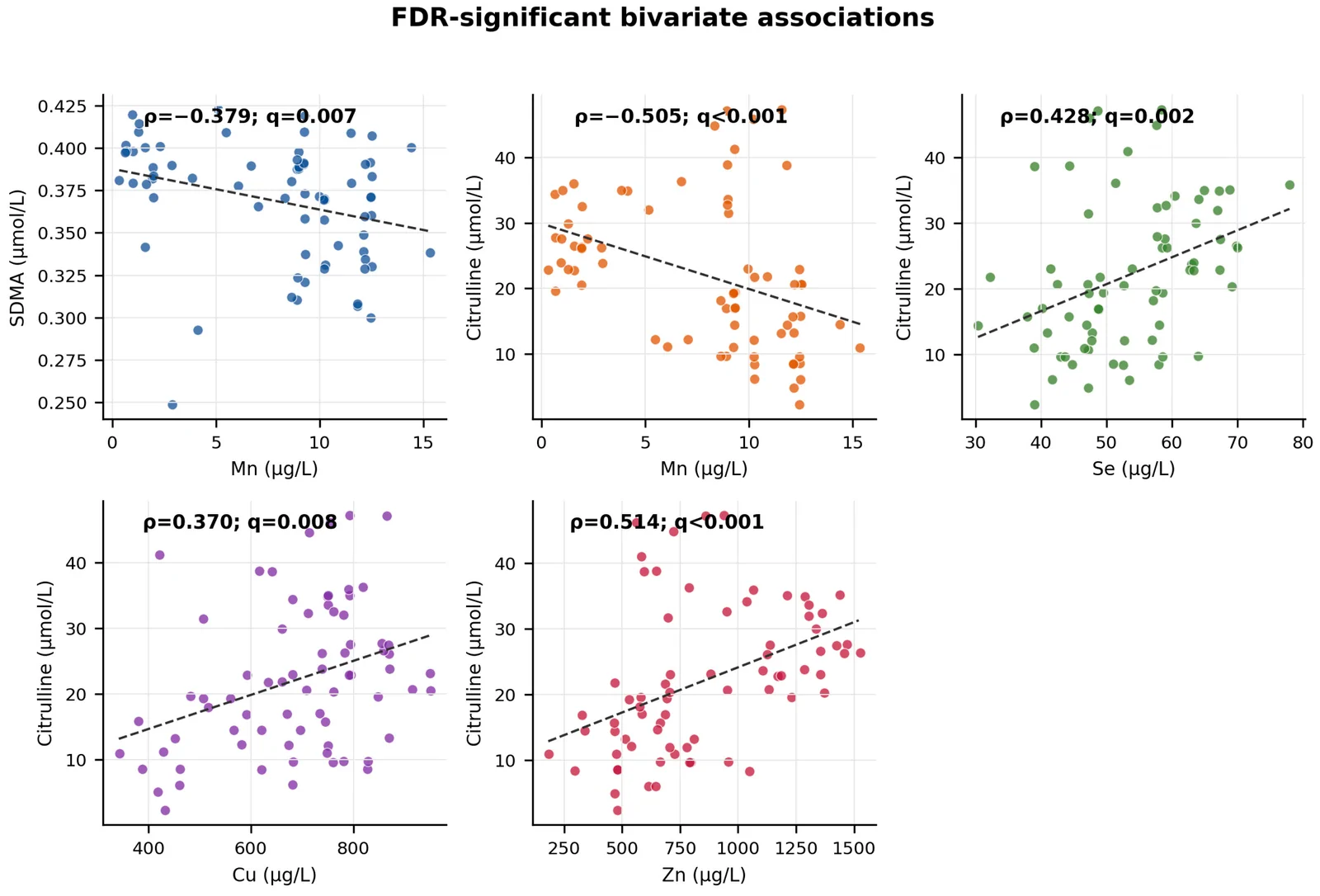
Scatterplots for the five FDR-significant bivariate associations. Points are individual participants; dashed lines are descriptive least-squares fits. Statistical inference is based on Spearman ρ and FDR-adjusted q values, not on the fitted lines.

**Table 1:** Participant characteristics and measured analyte distributions (*n* = 72).

Parameters	Unit	Mean ± SD	Median [IQR]	Range	External Comparison *
**Age**	years	41.57 ± 8.90	37.00 [31.00–48.25]	22.00–63.00	—
**BMI**	kg/m^2^	26.32 ± 7.14	27.06 [25.87–28.72]	22.64–42.80	—
**Mn**	µg/L	7.79 ± 4.35	9.12 [2.88–11.60]	0.32–15.36	4.7–18.3
**Se**	µg/L	53.46 ± 10.00	52.95 [47.20–59.41]	30.39–77.94	>70
**Cu**	µg/L	687.39 ± 149.96	722.44 [591.18–792.08]	341.68–949.90	850–1900
**Zn**	µg/L	853.22 ± 347.04	751.75 [585.78–1147.90]	181.74–1520.02	660–1100
**Sb**	µg/L	1.61 ± 0.33	1.61 [1.54–1.66]	0.51–3.54	0–3
**Mo**	µg/L	0.70 ± 0.21	0.65 [0.64–0.67]	0.59–1.74	0.3–2
**Tl**	µg/L	2.23 ± 0.33	2.25 [2.05–2.44]	1.26–2.88	0–2
**ADMA**	µmol/L	0.888 ± 0.244	0.900 [0.738–1.070]	0.350–1.330	—
**SDMA**	µmol/L	0.369 ± 0.035	0.380 [0.340–0.390]	0.250–0.420	—
**L-NMMA**	µmol/L	0.033 ± 0.020	0.030 [0.020–0.040]	0.000–0.100	—
**Citrulline**	µmol/L	22.11 ± 11.16	20.57 [13.01–30.28]	2.30–47.19	—

Mn (manganese), Se (selenium), Cu (copper), Zn (zinc), Sb (antimony), Mo (molybdenum), Tl (thallium), ADMA, asymmetric dimethylarginine; SDMA, symmetric dimethylarginine; L-NMMA, N-monomethyl-L-arginine. * External comparison values were obtained from ACGIH [43], except those for thallium and selenium, which were obtained from references [44,45]. These literature-based values are provided only for contextual comparison and are not study-specific diagnostic thresholds. Specifically, they are derived from occupational and toxicological guidance documents and do not constitute nutritional reference intervals for the general Turkish population.

**Table 2:** Associations between trace element concentrations and arginine-metabolism biomarkers (*n* = 72).

TE	ADMA	SDMA	L-NMMA	Citrulline
**Mn**	−0.116 (*p* = 0.33; q = 0.527)	−0.379 ** (*p* = 0.00102; q = 0.00713)	−0.294 (*p* = 0.0122; q = 0.0569)	−0.505 ** (*p* = 5.98 × 10^−6^; q < 0.001)
**Se**	0.045 (*p* = 0.707; q = 0.825)	0.188 (*p* = 0.114; q = 0.398)	0.126 (*p* = 0.293; q = 0.527)	0.428 ** (*p* = 0.000174; q = 0.00162)
**Cu**	−0.166 (*p* = 0.163; q = 0.414)	0.098 (*p* = 0.414; q = 0.579)	−0.056 (*p* = 0.642; q = 0.817)	0.370 ** (*p* = 0.00136; q = 0.00764)
**Zn**	−0.119 (*p* = 0.318; q = 0.527)	0.262 (*p* = 0.0259; q = 0.104)	0.173 (*p* = 0.147; q = 0.41)	0.514 ** (*p* = 3.83 × 10^−6^; q < 0.001)
**Sb**	−0.036 (*p* = 0.766; q = 0.825)	0.101 (*p* = 0.4; q = 0.579)	0.180 (*p* = 0.129; q = 0.402)	0.137 (*p* = 0.251; q = 0.527)
**Mo**	0.011 (*p* = 0.924; q = 0.958)	0.129 (*p* = 0.28; q = 0.527)	0.114 (*p* = 0.339; q = 0.527)	−0.066 (*p* = 0.582; q = 0.775)
**Tl**	0.001 (*p* = 0.994; q = 0.994)	−0.132 (*p* = 0.269; q = 0.527)	0.039 (*p* = 0.742; q = 0.825)	0.037 (*p* = 0.755; q = 0.825)

TE (trace elements), Mn (manganese), Se (selenium), Cu (copper), Zn (zinc), Sb (antimony), Mo (molybdenum), Tl (thallium), ADMA, asymmetric dimethylarginine; SDMA, symmetric dimethylarginine; L-NMMA, N-monomethyl-L-arginine. ** *p* < 0.01; all marked associations also remained significant after Benjamini–Hochberg FDR correction (q < 0.01).

**Table 3:** HC3-robust multivariable model summaries (*n* = 72).

Parameters	R^2^	Adjusted R^2^	HC3 Joint F	*p*
**ADMA**	0.168	0.047	1.48	0.174
**SDMA**	0.219	0.105	2.96	**0.005**
**L-NMMA**	0.187	0.069	1.28	0.266
**Citrulline**	0.271	0.165	4.07	**<0.001**

Each model included age, BMI, Mn, Se, Cu, Zn, Sb, Mo, and Tl. Joint tests used HC3-robust covariance estimates. ADMA, asymmetric dimethylarginine; SDMA, symmetric dimethylarginine; L-NMMA, N-monomethyl-L-arginine.

## Data Availability

The datasets analyzed during the current study are available from the corresponding author upon reasonable request.

## Supplementary Materials

The following supporting information can be downloaded at: https://www.mdpi.com/article/10.3390/jcm15218480/s1, Table S1: SDMA and citrulline across sample-based Mn quartiles; Table S2: Arginine-pathway biomarkers below and at or above each trace element’s sample median; Table S3: Complete HC3-robust multivariable regression coefficients.

## Abbreviations

ADMA	Asymmetric Dimethylarginine
BMI	Body Mass Index
CVD	Cardiovascular Disease
Cu	Copper
DDAH	Dimethylarginine Dimethylaminohydrolase
eGFR	Estimated Glomerular Filtration Rate
eNOS	Endothelial Nitric Oxide Synthase
FDR	False Discovery Rate
HDL-C	High-Density Lipoprotein Cholesterol
ICAM-1	Intercellular Adhesion Molecule-1
ICP-MS	Inductively Coupled Plasma Mass Spectrometry
IDF	International Diabetes Federation
L-NMMA	N-Monomethyl-L-Arginine
MetS	Metabolic Syndrome
Mn	Manganese
Mo	Molybdenum
MnSOD	Manganese Superoxide Dismutase
NAFLD	Non-Alcoholic Fatty Liver Disease
NO	Nitric Oxide
NOS	Nitric Oxide Synthase
ROS	Reactive Oxygen Species
Sb	Antimony
SDMA	Symmetric Dimethylarginine
Se	Selenium
T2DM	Type 2 Diabetes Mellitus
Tl	Thallium
VCAM-1	Vascular Cell Adhesion Molecule-1
Zn	Zinc
