# Supplementary material for: The Relationship Between Trace Element Levels and Arginines in Individuals with Metabolic Syndrome: An Exploratory Cross-Sectional Analysis of Endocrine and Nitric Oxide Metabolism
Source: J Clin Med. 2026 Oct 30;15(21):8480. doi: 10.3390/jcm15218480 (PMC13764270; doi:10.3390/jcm15218480)
Supplement: Supplementary file 1 [file jcm-15-08480-s001.zip › jcm-4516632-supplementary.pdf]

Table S1. SDMA and citrulline across sample-based Mn quartiles.

Parameters	Mn Q1	Mn Q2	Mn Q3	Mn Q4	Kruskal–Wallis	Q1 vs Q4 p*
**SDMA (μmol/L)**	0.390 [0.380–0.400] (n=19)	0.380 [0.370–0.390] (n=17)	0.370 [0.345–0.388] (n=18)	0.355 [0.330–0.378] (n=18)	H=10.28; p=0.016	0.003
**Citrulline (μmol/L)**	26.378 [23.353–28.798] (n=19)	31.944 [12.100–34.969] (n=17)	18.150 [12.402–21.780] (n=18)	13.915 [8.470–19.360] (n=18)	H=19.47; p<0.001	<0.001

The Mn cut points were 0.32 μg/L (minimum), 2.88 μg/L (Q1), 9.12 μg/L (median), 11.60 μg/L (Q3), and 15.36 μg/L (maximum). *Exploratory Mann–Whitney comparison without multiplicity adjustment.

Table S2. Arginine-pathway biomarkers below and at or above each trace element's sample median.

Trace (median)	Parameters	Below: n; median [IQR]	At/above: n; median [IQR]	p	q	Rank-biserial*
**Mn (9.120)**	ADMA	36; 0.905 [0.768–1.012]	36; 0.875 [0.698–1.105]	0.879	0.947	-0.022
**Mn (9.120)**	SDMA	36; 0.390 [0.378–0.400]	36; 0.365 [0.338–0.383]	0.007	0.050	-0.367
**Mn (9.120)**	L-NMMA	36; 0.030 [0.020–0.050]	36; 0.030 [0.020–0.040]	0.061	0.215	-0.253
**Mn (9.120)**	Citrulline	36; 27.044 [22.234–33.789]	36; 15.125 [9.680–20.570]	<0.001	0.001	-0.560
**Se (52.945)**	ADMA	36; 0.860 [0.698–1.062]	36; 0.915 [0.785–1.070]	0.386	0.583	0.120
**Se (52.945)**	SDMA	36; 0.370 [0.340–0.390]	36; 0.380 [0.367–0.400]	0.395	0.583	0.117
**Se (52.945)**	L-NMMA	36; 0.030 [0.020–0.040]	36; 0.030 [0.020–0.050]	0.471	0.651	0.098
**Se (52.945)**	Citrulline	36; 15.730 [10.890–20.873]	36; 26.317 [20.146–32.912]	<0.001	0.009	0.452
**Cu (722.440)**	ADMA	36; 0.950 [0.768–1.078]	36; 0.875 [0.705–1.025]	0.246	0.506	-0.160
**Cu (722.440)**	SDMA	36; 0.370 [0.340–0.390]	36; 0.380 [0.355–0.393]	0.511	0.651	0.090
**Cu (722.440)**	L-NMMA	36; 0.030 [0.020–0.040]	36; 0.030 [0.020–0.040]	0.991	0.991	-0.002
**Cu (722.440)**	Citrulline	36; 16.940 [10.890–22.899]	36; 24.986 [18.755–32.912]	0.010	0.053	0.356

**Zn (751.750)**	ADMA	36; 0.930 [0.768–1.105]	36; 0.885 [0.705–1.025]	0.295	0.506	-0.144
**Zn (751.750)**	SDMA	36; 0.370 [0.340–0.383]	36; 0.390 [0.370–0.400]	0.012	0.058	0.341
**Zn (751.750)**	L-NMMA	36; 0.030 [0.020–0.040]	36; 0.030 [0.020–0.050]	0.196	0.463	0.175
**Zn (751.750)**	Citrulline	36; 15.730 [10.890–20.873]	36; 26.317 [20.570–32.912]	<0.001	0.007	0.477
**Sb (1.610)**	ADMA	31; 0.880 [0.755–1.060]	41; 0.900 [0.710–1.070]	0.927	0.962	0.013
**Sb (1.610)**	SDMA	31; 0.370 [0.340–0.390]	41; 0.380 [0.350–0.400]	0.560	0.681	0.081
**Sb (1.610)**	L-NMMA	31; 0.020 [0.020–0.040]	41; 0.030 [0.020–0.040]	0.083	0.258	0.237
**Sb (1.610)**	Citrulline	31; 19.360 [10.890–26.862]	41; 22.869 [14.520–32.670]	0.135	0.377	0.208
**Mo (0.650)**	ADMA	26; 0.895 [0.762–1.028]	46; 0.915 [0.732–1.093]	0.792	0.897	0.038
**Mo (0.650)**	SDMA	26; 0.370 [0.333–0.390]	46; 0.380 [0.360–0.400]	0.289	0.506	0.151
**Mo (0.650)**	L-NMMA	26; 0.025 [0.020–0.037]	46; 0.030 [0.020–0.050]	0.060	0.215	0.265
**Mo (0.650)**	Citrulline	26; 19.965 [11.193–23.625]	46; 21.719 [14.520–31.430]	0.308	0.506	0.146
**Tl (2.255)**	ADMA	36; 0.915 [0.725–1.083]	36; 0.885 [0.748–0.992]	0.496	0.651	-0.094
**Tl (2.255)**	SDMA	36; 0.380 [0.360–0.400]	36; 0.375 [0.340–0.390]	0.198	0.463	-0.176
**Tl (2.255)**	L-NMMA	36; 0.030 [0.020–0.043]	36; 0.030 [0.020–0.040]	0.801	0.897	-0.035
**Tl (2.255)**	Citrulline	36; 20.449 [12.100–26.681]	36; 22.385 [14.520–32.912]	0.284	0.506	0.147

Mann–Whitney U tests were used; q values were calculated using the Benjamini–Hochberg correction across 28 comparisons. *Positive rank-biserial values indicate higher biomarker ranks in the at-or-above-median group. Ties at the median explain unequal group sizes for some elements.

Table S3. Complete HC3-robust multivariable regression coefficients.

Parameters	Predictor	B	95% CI	Standardized β	p	VIF
**ADMA**	Age	-0.00198	-0.00832 to 0.00436	-0.083	0.535	1.18
**ADMA**	BMI	-0.00426	-0.02399 to 0.01546	-0.055	0.667	1.22

**ADMA**	Mn	-0.02014	-0.04921 to 0.00892	-0.360	0.171	4.30
**ADMA**	Se	0.00011	-0.00967 to 0.00989	0.005	0.982	2.72
**ADMA**	Cu	0.00004	-0.00045 to 0.00052	0.023	0.879	1.84
**ADMA**	Zn	-0.00032	-0.00072 to 0.00009	-0.453	0.123	3.98
**ADMA**	Sb	-0.12560	-0.30416 to 0.05296	-0.168	0.165	1.04
**ADMA**	Mo	-0.22113	-0.59027 to 0.14801	-0.195	0.236	1.08
**ADMA**	Tl	0.06046	-0.12689 to 0.24781	0.083	0.521	1.10
**SDMA**	Age	-0.00013	-0.00129 to 0.00103	-0.037	0.826	1.18
**SDMA**	BMI	-0.00248	-0.00499 to 0.00002	-0.224	0.052	1.22
**SDMA**	Mn	-0.00475	-0.00955 to 0.00004	-0.587	0.052	4.30
**SDMA**	Se	-0.00050	-0.00183 to 0.00083	-0.142	0.454	2.72
**SDMA**	Cu	0.00003	-0.00005 to 0.00010	0.112	0.485	1.84
**SDMA**	Zn	-0.00004	-0.00011 to 0.00004	-0.352	0.362	3.98
**SDMA**	Sb	0.01123	-0.02920 to 0.05165	0.104	0.581	1.04
**SDMA**	Mo	-0.02841	-0.15838 to 0.10156	-0.173	0.664	1.08
**SDMA**	Tl	-0.00524	-0.03465 to 0.02417	-0.050	0.723	1.10
**L-NMMA**	Age	-0.00017	-0.00071 to 0.00038	-0.085	0.549	1.18
**L-NMMA**	BMI	-0.00022	-0.00153 to 0.00109	-0.035	0.739	1.22
**L-NMMA**	Mn	-0.00253	-0.00488 to -0.00019	-0.559	0.035	4.30
**L-NMMA**	Se	-0.00053	-0.00135 to 0.00029	-0.269	0.202	2.72
**L-NMMA**	Cu	-0.00003	-0.00008 to 0.00002	-0.232	0.227	1.84
**L-NMMA**	Zn	3.22e-06	-0.00003 to 0.00003	0.057	0.827	3.98
**L-NMMA**	Sb	0.00392	-0.02519 to 0.03304	0.065	0.789	1.04
**L-NMMA**	Mo	-0.00715	-0.04160 to 0.02729	-0.078	0.679	1.08
**L-NMMA**	Tl	0.00534	-0.01147 to 0.02215	0.091	0.528	1.10
**Citrulline**	Age	0.13292	-0.09571 to 0.36154	0.121	0.250	1.18
**Citrulline**	BMI	0.28921	-0.92356 to 1.50198	0.082	0.635	1.22
**Citrulline**	Mn	-0.49400	-1.64497 to 0.65697	-0.192	0.394	4.30
**Citrulline**	Se	0.12411	-0.27019 to 0.51841	0.111	0.532	2.72
**Citrulline**	Cu	0.01617	-0.00718 to 0.03951	0.217	0.171	1.84
**Citrulline**	Zn	0.00313	-0.01006 to 0.01633	0.097	0.636	3.98
**Citrulline**	Sb	0.89906	-2.22263 to 4.02076	0.026	0.567	1.04
**Citrulline**	Mo	-7.28645	-16.36875 to 1.79586	-0.140	0.114	1.08
**Citrulline**	Tl	2.63026	-5.44016 to 10.70069	0.079	0.517	1.10

All models included the nine listed predictors simultaneously (n=72). Confidence intervals and p values were calculated using HC3-robust standard errors. Model-level statistics are shown in Table 3.
